# 

**Published:** 1921-04-30

**Authors:** 


April 30, 1921] [Price Twopence

### The Hospital

The Workers' Newspaper of Administrative Medicine and Institutional Life, Administration, National Insurance and Health.

CONTENTS

The Hospital Profession ... ... ... ... ... 75

An Industrial Clinic ... ... ... ... ... ... 75

A Training for Hospital Executives ... ... ... 76

The Fight against Disease ... ... ... ... ... 76

Notes of the Week ... ... ... ... ... 77
A Sad Decision—A Centre of British Medicine—A Medical Knighthood—Hospitals and Insured Patients—Psycho-Analysis—" A Slave to the Hospital "—The Hospital Pool—Death of Mr. Edmund Forster—The Rochdale Infirmary Secretary—The Government and Hospital Research—Headache—Co-operation in Manchester—The Hospital Family.

Research on the Common Fevers ... ... ... 79

The Healthy Mind ... ... ... ... ... ... 80

Some International Health Questions and the League of Nations ... ... ... ... ... 81
An Address to the Kensington Branch of the League of Nations Union.

The Public Well-Being ... ... ... ... ... ... 83
Popular Health Methods—The Illegitimate Child—Children and Crime.

The Cirencester Experiment ... ... ... ... 85
A Paying Hospital for all Classes and all Incomes.

The Editor's Letter-Box .. ... ... ... ... 86

" The Practice of Birth-Control "—Waist-Bands for Fat Men.

The Matrons' and Sisters' Department ... ... 87
The Pay of the Nurse.—VIII. The Outlook for the Future.

Round the Hospitals ... ... ... ... ... 88

Matrons' and Sisters' Appointments ... ... 89

Parliament and the Professions ... ... ... 90

Annual Meetings of Hospitals ... ... ... ... 91

The Appeal of the Invalid Child ... ... ... 91

The College of Nursing (Limited by Guarantee) 92
What the Local Centres are Doing.

